# Enhanced Immune Response Against Echinococcus Granulosus Through a CTLA-4/B7 Affinity-Based Vaccine

**DOI:** 10.3390/vaccines12121440

**Published:** 2024-12-20

**Authors:** Yuejie Zhu, Yueyue He, Ziyue Yin, Na Chen, Xingxing Qi, Jianbing Ding, Yujiao Li, Fengbo Zhang

**Affiliations:** 1 Reproductive Medicine Center, The First Affiliated Hospital of Xinjiang Medical University, Urumqi 830054, China; zhuyuejie_1216@163.com; 2 Department of Immunology, School of Basic Medical Sciences, Xinjiang Medical University, Urumqi 830011, China; aaayou8biou@outlook.com (Y.H.); yyyinziyue@outlook.com (Z.Y.); dingjb1234@aliyun.com (J.D.); 3 School of Public Health, Guilin Medical University, Guilin 541100, China; 4 Clinical Laboratory Center, The First Affiliated Hospital of Xinjiang Medical University, Urumqi 830054, China; cnlhn520@163.com (N.C.); xjmuqxx@163.com (X.Q.); 5 State Key Laboratory of Pathogenesis, Prevention, Treatment of Central Asian High Incidence Diseases, The First Affiliated Hospital of Xinjiang Medical University, Urumqi 830054, China; 6 Post-Doctoral Research Station of the Clinical Medicine, The First Affiliated Hospital of Xinjiang Medical University, Urumqi 830054, China

**Keywords:** CE, vaccine, epitope, immunoinformatics, CTLA-4, molecular docking

## Abstract

**Background:** Echinococcosis is a zoonotic infectious disease that poses a significant threat to the health of individuals living in rural regions. While vaccination represents a potential strategy for disease prevention, there is currently no effective vaccine available for humans to prevent cystic echinococcosis (CE). This study aimed to design a novel multi-epitope vaccine (MEV) against Echinococcus granulosus for human use, employing immunoinformatics methods. **Methods:** We identified core epitopes from two key antigens, EgA31 and EgG1Y162, and integrated them into the immunoglobulin variable region of CTLA-4 (CTLA-4lgV) to create the *CVE31-162* vaccine construct. The secondary and tertiary structures of the *CVE31-162* were established using bioinformatics methods. The interaction between the *CVE31-162* and B7 molecules was assessed through molecular dynamics simulations. Finally, both in vitro and in vivo experiments were conducted to validate the effectiveness of the *CVE31-162* against the immunological effects of Echinococcus granulosus. **Results:** Bioinformatics analysis indicated that *CVE31-162* exhibits favorable antigenicity, stability, and non-allergenicity. Furthermore, *CVE31-162* demonstrated a stable three-dimensional structural model. Molecular docking (MD) and molecular dynamics simulations (MDS) revealed a strong binding affinity between *CVE31-162* and B7 molecules. Immune simulation results suggested that the vaccine elicits robust humoral and cell-mediated immune responses. Both in vitro and in vivo experiments demonstrated that immunized mice exhibited significantly elevated levels of antigen-specific antibodies and enhanced lymphocyte proliferation compared to the control group. **Conclusions:** *CVE31-162*, which is based on the interaction between CTLA-4 and B7, represents a promising multi-epitope vaccine for Echinococcus granulosus.

## 1. Introduction

Echinococcosis is a zoonotic disease that affects humans and is caused by the larval forms (metacestodes) of certain cestode species within the Echinococcus genus. Cystic echinococcosis (CE) is associated with Echinococcus granulosus (*E. granulosus*), whereas alveolar echinococcosis (AE) results from *E. multilocularis*. Additionally, polycystic variations are attributed to either *E. vogeli* or *E. oligarthrus* [1]. Currently, molecular research categorizes *E. granulosus* into several genotypes (G1–G10). Among these, *E. granulosus* sensu stricto, primarily represented by the G1 genotype, is the most prevalent population closely associated with human diseases [2]. In the natural life-cycle of *E. granulosus*, definitive hosts primarily include dogs and other canids, whereas ungulates—such as sheep, goats, pigs, and horses—act as intermediate hosts. These intermediate hosts harbor the metacestode stage, which can also develop in a range of other mammals, including marsupials, hares, rabbits, rodents, carnivores, nonhuman primates, and even humans [3]. Despite the availability of control measures, CE continues to pose a major public health challenge in numerous areas across both the northern and southern hemispheres. In 2015, the Foodborne Disease Burden Epidemiology Reference Group (FERG), which is part of the World Health Organization, conducted a comprehensive evaluation of echinococcosis. This assessment revealed that the disease is responsible for approximately 19,300 fatalities, highlighting its severe impact on public health. Additionally, the study estimated that echinococcosis accounts for around 871,000 disability-adjusted life-years (DALYs), a measure that reflects the overall burden of the disease in terms of both premature mortality and loss of healthy life. Within the framework of One Health, WHO actively developed echinococcosis control programs with the World Organization for Animal Health (OIE).

In terms of symptoms, CE is a chronic disease with a long incubation period. At the early stage of infection, infection was asymptomatic and difficult to detect [4]. WHO listed echinococcosis as a neglected tropical disease (World Health Statistics 2015, https://www.who.int/publications/i/item/9789240694439, accessed on 16 April 2022). The most common definitive host for *E. granulosus* worldwide is dogs [5]. Given the close relationship between dogs and humans, the likelihood of humans being infected with *E. granulosus* is increased. In the context of, the treatment approach is determined by the type of cyst, as classified by the WHO-IWGE ultrasound classification [6]. This classification takes into account various factors, including the size and location of the cyst, the presence or absence of complications, and the availability of medical resources and equipment. From a clinical perspective, surgery remains the gold standard for the treatment of echinococcosis [7,8]. However, the release of hydatid fluid, which is rich in protoscoleces, during surgical interventions or the “PAIR” (puncture, aspiration, injection, and re-aspiration) procedure for cystic echinococcosis is a significant factor contributing to the recurrence of cysts [9]. In addition to this, when the parasites are transferred to other organs, the therapeutic effect of surgery is not ideal [10]. Therefore, it is essential to take the necessary precautions.

The basic principles for the prevention and control of infectious diseases are to manage the source of infection, cut off the transmission route, and protect the susceptible population [11]. However, a series of problems such as drug resistance and environmental pollution are caused by using a large number of deworming drugs [12]. Therefore, in addition to personal hygiene practices, preventive immunization is also needed. This can not only reduce the damage caused by surgery and drugs to humans, but it is also a relatively cost-effective preventive measure for susceptible people [13]. In contemporary times, the development of vaccines for humans has progressively transitioned from traditional methods to innovative approaches. In comparison to conventional vaccine development processes, epitope-based prophylactic vaccines represent a more cost-effective and time-efficient strategy [14]. The successful identification and selection of dominant epitopes from key vaccine candidate antigens (VCAs) are particularly crucial for the construction of epitope-based vaccines. In this context, the in silico modeling of vaccines offers an effective approach [15]. Previous studies on *E. granulosus* have identified several vaccine candidate antigens (VCAs), including Eg95, EgA31, EgDf1, EgAg5, and EgG1Y162 [16]. These findings offer multiple options for the development of a multi-epitope vaccine against *E. granulosus*.

EgA31 and EgG1Y162 are two proteins that can be used as VCAs, in which the former can be expressed in the adult stage and the latter can be expressed in the larval stage [17,18]. EgA31, a fibrillar protein identified by Fu et al. as one of the major antigens of *E. granulosus* [19,20], is predominantly found in the microhairs of the pre-adult stage. This finding is entirely consistent with its presence in the microhairs of the still invaginated protoscolex, showing no sharp contrast with that. Preliminary studies suggest that all domains of EgA31 contribute to the immune response elicited by the parasite. Furthermore, in vivo evidence indicates that this peptide stimulates immune responses in dogs, at both the humoral and cellular level, shortly after infection [19].

EgG1Y162 protein was a new antigen gene amplified from fine-grained *E. granulosus* by Cao [18]. After an in-depth bioinformatics analysis of the antigen by Zhang et al. [21], it was found that the antigen had a T-cell epitope, B-cell epitope and T-B epitopes with strong immunogenicity. Zhao [22] showed that EgG1Y162 can be used as a VCA through epitopes analysis. The immunogenicity of a single-epitope vaccine is low, so we hope to build a multi-epitope vaccine.

CTLA-4 (cytotoxic T-lymphocyte-associated protein 4), also referred to as CD152, is expressed on the surface of T cells. Similar to CD28, CTLA-4 interacts with ligands present on the surface of antigen-presenting cells (APCs), such as dendritic cells, primarily binding to B7 (including B7-1 and B7-2) [23,24]. GGGGS is a commonly used flexible linker in protein engineering and antibody design [25]. Research indicates that the extracellular domain of CTLA-4 (CTLA-4lgV) is linked to the vaccine antigen via (GGGGS)_3_. After the fusion of antigens with CTLA-4, smaller antigens showed stronger humoral immunity [26]. Therefore, we adopted the method of selecting the dominant epitopes of VCAs to build a multiple epitopes vaccine, which not only reduced the size of the antigen, but also retained the immunogenicity of the antigen.

In this study, we selected EgA31 and EgG1Y162 proteins to predict their B- and T-cell epitopes by immunoinformatics, and selected the dominant epitope for connection to build the epitope fusion vaccine. At the same time, we connected the immunoglobulin variable region of CTLA-4 (CTLA-4lgV) to the epitope fusion vaccine to improve the immunogenicity of the vaccine, and finally form the multi-epitope vaccine *CVE31-162*. This multi-epitope vaccine, *CVE31-162*, is expected to provide a promising approach for developing effective immunoprophylactic strategies against *Echinococcus granulosus*, contributing to the prevention and control of echinococcosis.

## 2. Materials and Methods

### 2.1. Obtaining the Acid Sequence, Signal Peptide, and Transmembrane Domains of Proteins

The amino acid sequences for EgA31 and EgG1y62 were retrieved from the National Center for Biotechnology Information (NCBI) online database (https://www.ncbi.nlm.nih.gov/genbank/. accessed on 16 April 2022). To analyze the transmembrane domains of these proteins, we utilized the TMHMM platform, which is accessible online (https://services.healthtech.dtu.dk/service.php?TMHMM-2.0, accessed on 16 April 2022). This software serves as a tool to forecast transmembrane helices by employing a Markov model approach.

It integrates the properties of transmembrane hydrophobicity, charge distribution, helix length, and the topological constraints of membrane proteins. This software can predict not only the transmembrane regions, but also the inner and outer regions of the membrane in their entirety [27]. The signal peptide was analyzed by the online server signalP-6.0 (https://services.healthtech.dtu.dk/service.php?SignalP, accessed on 16 April 2022).

### 2.2. The Prediction of T-Cell Epitopes of Proteins

The T-cell antigen receptor (TCR) enables T cells to recognize antigens by identifying major histocompatibility complex (MHC) molecules, referred to as human leukocyte antigens (HLA) in humans [28]. T lymphocytes are classified as either CD8+ or CD4+, depending on their specific immune roles [29].

Due to the fact that the vaccine was an exogenous antigen, which stimulates immunity in human body through HLA-II pathway [30]. MHC II molecules play a crucial role in the presentation of antigens to T cells and in the immune response. A powerful tool called NetMHCIIpan version 4.0, based on artificial neural networks, has been documented as effective for predicting CD4+ T-cell epitope peptides bound by MHC II. This prediction tool utilized parameters, such as binding affinity (BA), and scores from eluted ligand mass spectrometry (EL) to identify epitope peptides of various lengths [31]. Next, we utilized the “T-cell epitope prediction tool” and “B-cell epitope prediction tool”, available on the IEDB (tools.iedb.org/mhcii/, accessed on 17 April 2022) platform, to predict epitopes, which is designed to identify potential T-cell epitopes based on binding affinity predictions, MHC class II or MHC class I binding predictions. We chose the high-frequency alleles HLA-DRB1*03:01, HLA-DRB1*07:01 and HLA-DRB1*15:01 in an ethnic Uyghur population in the Xinjiang region of China as the parameter [22]. The predicted results were ranked according to grades and scores. The higher the score, the lower the grade. We include the top 50 epitopes with grades less than 3.7 and scores greater than 3.8 for further analysis. Finally, according to the prediction results of the two, we selected the top 50 epitopes, respectively. After selecting their core epitopes, we use the overlapping core epitopes as the advantageous epitopes of EgA31 and EgG1y62.

### 2.3. Prediction of Protein B-Cell Epitopes

B-cell epitopes are categorized into linear and conformational types [32]. In order to improve efficiency, we chose two online prediction software, IEBD (http://tools.iedb.org/bcell/, accessed on 17 April 2022) and ABCpred (https://webs.iiitd.edu.in/raghava/abcpred/index.html, accessed on 17 April 2022), with different algorithms [33]. This tool aids in assessing the linear conservancy of epitopes and supports epitope-based vaccine development [34]. They were based on physicochemical-based algorithms and machine learning-based algorithms, respectively. We used these two different online software to predict the B-cell linear epitopes of EgA31 and EgG1y62.

### 2.4. Construction of the Multi-Epitope Vaccine CVE31-162

Based on Roong Dong’s research [35], we chosen some linker peptides to construct our vaccine model. Linker-AAT is used to connect T-cell epitopes and used the linker-KK to connect B-cell epitopes. According to the research of Pourseif et al. [16], the Gly-Pro-Ser-Leu (GPSL) linker is used to connect the T- and B-cell epitopes. An application (GGGGS)_3_ sequence is used to connect CTLA-4lgV to the T epitopes of EgA31. Eventually, we formed a multi-epitope vaccine *CVE31-162.*

### 2.5. Prediction of Physicochemical Properties of Vaccine CVE31-162

The ProtParam online tool (www.web.expasy.org/protparam/, accessed on 18 April 2022) was utilized to predict the physicochemical properties of *CVE31-162*. This included assessments of molecular weight, isoelectric point (pI), amino acid composition, atomic structure, extinction coefficient, half-life, instability index, aliphatic index, and the grand average of hydropathicity (GRAVY) score, all of which were based on the primary sequence of the epitope.

### 2.6. Analysis Antigenicity and Allergenicity of Vaccine CVE31-162

After completing the construction of the vaccine, the antigenicity and allergenicity of *CVE31-162* was evaluated. The online software VaxiJen 2.0 (http://www.ddg-pharmfac.net/vaxijen/VaxiJen/VaxiJen.html, accessed on 20 April 2022) was used to analyze the antigenicity of vaccines. The online software AllerTOP2.0 (http://www.ddg-pharmfac.net/AllerTOP/, accessed on 20 April 2022) to predict allergenicity. This tool predicted antigenicity and allergenicity from the antigenic epitope sequence dataset [27].

### 2.7. Secondary Structure Prediction of CVE31-162

The secondary structure of *CVE31-162* was predicted using the SOMPA online tool (https://npsa-prabi.ibcp.fr/cgi-bin/npsa_automat.pl?Page=npsa_sopma.html, accessed on 21 April 2022), analyzing various elements such as alpha helices, extended strands, beta turns, and random coils.

### 2.8. The Prediction and Quality Validation of the Tertiary Structure of the Vaccine CVE31-162

We used the online software RoseTTAFold 2.0 (https://robetta.bakerlab.org/submit.php, accessed on 21 April 2022) to predict the tertiary structure of *CVE31-162*. RoseTTAFold 2.0 not only produces a two-track network but also extends to a three-track network, thereby establishing a closer connection between residue–residue distance and orientation, as well as sequence and atomic coordinates. Additionally, the network can rapidly model protein–protein complexes using only sequence information [36]. Used SWISS-MODEL’s structural evaluation service (https://swissmodel.expasy.org/, accessed on 21 April 2022) to verify the quality of the tertiary structure constructed by vaccines. The SWISS-MODEL relies on the weekly pre-release sequences from the PDB, enabling us to consistently assess and enhance the server’s performance [37].

### 2.9. Molecular Docking for CVE31-162 and B7

#### 2.9.1. Acquirement of B7 Molecules

The 3D structure models of B7 molecules were edited using DS software 2019 based on data from the PDB database (https://www.rcsb.org/, accessed on 21 April 2022), resulting in the final tertiary structures of B7-1 and B7-2 molecules for docking.

#### 2.9.2. The Docking of Vaccine CVE31-162 with B7

The molecular docking of *CVE31-162* and B7 was executed by LZerD Web Server. LZerD employs a soft protein surface representation utilizing 3D Zernike descriptors and investigates the binding pose space through geometric hashing. This approach demonstrates effective performance in molecular docking. The three-dimensional structures of the two were entered into the LZerD Web Server as a ligand and receptor. Then, obtained a variety of docking conformations. At the same time, we would be comprehensively ranked according to the scores of DFIRE, GOAP, and ITScore. The higher the ranking, the better the quality of docking [38].

### 2.10. Molecular Dynamics and Principal Component Analysis

Molecular dynamics (MD) is a comprehensive technology that combines physical chemistry and mathematics. MD simulation (MDS) simulates the motion of the molecular system based on Newtonian mechanics [39]. In order to explore the stability of the docking complex, MDS was carried out using Gromacs2022.3, with small molecule preprocessing performed through AmberTools22 and Gaussian 16W. The GAFF force field was utilized along with RESP charges, and the Amber99sb-ildn force field was applied for the protein components. The simulations were performed at a temperature of 300 K and a pressure of 1 Bar, utilizing TIP3P water along with Na+ ions to ensure neutrality. For energy minimization, the steepest descent approach was employed, followed by NVT and NPT equilibration phases, each lasting 100 ps. A production run lasting 100 ns was executed using 2 fs time steps. The trajectory analysis featured calculations for RMSD, RMSF, the radius of protein rotation, and MMGBSA free energy assessments [40].

The conformational state of proteins in the natural environment had many possibilities [41]. In order to explore the conformation state of the vaccine, principal component analysis (PCA) of the atoms in the complex system was carried out [42]. PCA is a statistical method. The trajectory of MD were projected to the principal modes through Gromacs, and the most important critical principal component 1 (PC1) and principal component 2 (PC2) were visualized [43].

### 2.11. Immune Simulation

Immune simulation of *CVE31-162* was performed using the online software C-ImmSim (https://kraken.iac.rm.cnr.it/C-IMMSIM/, accessed on 23 April 2022) [44]. C-ImmSim predicts immunologic interactions using a position-specific scoring matrix (PSSM) [45]. In this simulation, HLA was chosen based on the host’s heterozygous HLA combinations: HLA-A (HLA-A*11:01, HLA-A*02:01), HLA-B (HLA-B*15:01, HLA-B*35:01), and HLA-DR (HLA-DRB1*07:01, HLA-DRB1*15:01). At the same time, the server modelled three distinct sections corresponding to three varying anatomical regions in mammals: the bone marrow, the thymus, and the lymphatic system. The three specific intervals used were 1, 84, and168. Ultimately, the software’s settings were returned to their default configurations, with the random seed for the simulation parameter established at 12,345, the volume of the simulation defined as 50, and the total simulation steps configured to 1050.

### 2.12. In Vitro Experiment

In this research, we examined a cohort of 25 patients (adults aged 18–60) who had been diagnosed with cystic echinococcosis at the Infection Department of the First Affiliated Hospital of Xinjiang Medical University. We also recruited 12 healthy adults (aged 18–60) to serve as a control group. All patients included in the study complied with the Chinese protocols for the diagnosis and treatment of cystic echinococcosis (CE), and we excluded any other health issues that might interfere with the experimental outcomes. We collected 4 mL of peripheral blood from each participant, both patients and healthy controls, from which serum was extracted for Western blotting (WB) analysis. Furthermore, peripheral blood mononuclear cells (PBMCs) from the patients were separated using Ficoll Paque, and subsequent in vitro cell experiments were performed.

#### 2.12.1. Detecting the Antigenicity of CVE31-162 by WB

A Western blot (WB) experiment was conducted to assess the antigenicity of *CVE31-162*. The loading buffer containing *CVE31-162* (1 µg/µL) was separated via SDS-PAGE, and the protein was transferred onto a PVDF membrane. The membrane was blocked with 5% skimmed milk powder solution and incubated overnight at 4 °C with diluted human serum (1:200) from CE patients (*n* = 10) and healthy controls (*n* = 10). Afterward, it was treated with an HRP-conjugated rabbit anti-human IgG secondary antibody (1:5000 dilution) for one hour at room temperature. Finally, the target protein band was visualized using an ECL detection kit (BL523B, Biosharp, Beijing, China) and exposed on Amersham hyperfilm (GE Healthcare, Beijing, China).

#### 2.12.2. Detecting the Immunogenicity of CVE31-162 by ELISA

An enzyme-linked immunosorbent assay (ELISA) was conducted to evaluate the immunogenicity of *CVE31-162*. PBMCs were isolated from patients and stimulated with either sterile PBS buffer (control group, *n* = 6) or *CVE31-162* protein (*CVE31-162* group, n = 6) for 48 h at 37 °C in a humidified incubator with 5% CO_2_. The supernatant from the cell cultures was collected to measure IFN-γ and IL-4 levels using ELISA. Separate ELISA plates were prepared for each cytokine, with two replicates per sample. The subsequent procedures followed the kit protocols. The ELISA assays utilized the human IFN-γ ELISA kit (SEKH-0046, Solarbio Science & Technology Co., Ltd., Beijing, China) and the human IL-4 ELISA kit (SEKH-0011, Solarbio Science & Technology Co., Ltd., Beijing, China).

#### 2.12.3. Detecting the Immunogenicity of CVE31-162 by ELISPOT

PBMCs from both the control group (*n* = 6) and the *CVE31-162* group (n = 6) were plated into ELISPOT plates, with two replicates per sample. Corresponding antigens were added, and the plates were incubated for 48 h in a cell incubator. The remaining steps followed the kit instructions, and total IgG antibody-secreting cells (ASCs) were detected at the conclusion of the procedure. The ELISpot flex-human IgG (ALP) Kit (3850-2A, Mabtech, Stockholm, Sweden) was used for the ELISPOT experiment.

### 2.13. In Vivo Experiment

In this study, 8-week-old SPF-grade BALB/C mice from Xinjiang Medical University’s Animal Experiment Center were divided into two groups: the *CVE31-162* group (*n* = 6), receiving intraperitoneal injections of *CVE31-162* with Freund’s adjuvant (50 µg/mouse), and the control group (*n* = 6), receiving PBS with Freund’s adjuvant (50 µg/mouse). Immunizations occurred biweekly for three sessions, using a complete Freund’s adjuvant (CFA) for the first dose and an incomplete Freund’s adjuvant (IFA) for subsequent boosters. Two weeks post-final immunization, serum and splenocytes were collected from six euthanized mice per group for flow cytometry (FCM) and ELISA analysis.

#### 2.13.1. Analysis of Specific CD4+ and CD8+ T-Cell Levels by FCM

Two weeks post-immunization, splenocytes were collected and counted using a hemocytometer. The cell concentration was adjusted to 2 × 10^6^ cells/mL, and the suspension was incubated with anti-CD3 (APC) and anti-CD4 (FITC) monoclonal antibodies for 20 min. CD4+ T cells were identified as CD3+CD4+ cells via flow cytometry using the FACSLyric from BD, with data analyzed using the FlowJo 10.8.1 software.

#### 2.13.2. Analysis of the Specific Antibody Level by ELISA

To assess *CVE31-162* antigenicity, ELISA was used to track anti-*CVE31-162* antibody levels in an animal model. Blood was drawn from the retro-orbital sinus before each immunization, placed in coagulation tubes, and centrifuged for serum collection. Microplates were coated with 6 μg/mL *CVE31-162* protein in bicarbonate buffer (pH 9.6), blocked with 5% milk in PBST, and incubated with 1:100 diluted mouse serum (100 μL) at 37 °C for 30 min. After PBST washes, an HRP-labeled secondary antibody (1:1000) was added, and absorbance at 450 nm was measured using a microplate reader.

#### 2.13.3. Detection of the Macrophages Level in Spleen and Liver with FCM

A 40% Percoll solution was used to separate mouse liver leukocytes. The suspension of splenocytes and liver leukocytes were incubated with monoclonal antibodies against CD45 (APC), F4/80 (FITC), and CD11b (APC-Cy7) for 20 min. Subsequently, CD45+ cells were gated using FlowJo software, and finally, F4/80+ CD11b+ was gated to identify macrophages. The flow cytometer used in this study was BD FACSLyric.

#### 2.13.4. Assessing the Uptake of CVE31-162 by Macrophages

The splenocytes were cultured in a medium containing 1% glutamine, 1% double antibody, 10% fetal bovine serum, and 88% DMEM at 37 °C and 5% CO_2_ overnight. Subsequently, a His-tagged *CVE31-162* recombinant protein was added to the cultured cells (final concentration 10 μg/mL) and incubated at 37 °C for 4 h. The cell suspension was transferred to a 15 mL centrifuge tube and centrifuged at 1200 rpm for 5 min, and the supernatant was removed. The cells were then resuspended in chilled PBS. The cells were then incubated in the dark at 4 °C for 20 min with monoclonal antibodies against CD45 (APC), F4/80 (FITC), CD11b (APC-Cy7), and an anti-His tag (PE) to assess the macrophage uptake of the *CVE31-162* protein.

#### 2.13.5. Assessment of CVE31-162’s Potential Protective Effects

To evaluate the protective effects of *CVE31-162* against *E. granulosus* protoscolices infection, mice were divided into two groups. After 22 weeks of infection, the mice were dissected, and the hepatic cyst weights were recorded.

## 3. Results

### 3.1. Detection of EgA31 and EgG1Y162 by Bioinformatics Method

The NCBI accession numbers and protein sequences for the EgA31, EgG1Y162, and CTLA-4 proteins are provided in Supplementary Material S1. The online server TMHMM showed that neither proteins EgA31 nor EgG1y62 had transmembrane domains (Figure S1). The online server signal P-6.0 showed that there was no signal peptide in EgA31 (Figure S2A). EgG1Y162 had a signal peptide, and its sequence was MVLRFCLILLATSVIA (Figure S2B). The signal peptide sequence was deleted, and the protein was directed to the appropriate subcellular organelles [46], so we removed the signal peptide sequence in subsequent predictions and analysis.

### 3.2. Prediction of Protein T-Cell and B-Cell Epitopes

In order to improve accuracy, we used two different online prediction software, IEDB and NetMHCIIpan 4.0 Server, to predict the T-cell epitopes of EgA31 and EgG1y62, respectively. Finally, the T-cell epitopes of EgA31 and EgG1y62 are shown in Table 1.

The linear B-cell epitopes in the online server ABCpred were predicted based on the artificial neural network. The IEDB server predicted the linear B-cell epitopes via physicochemical-based algorithms. By predicting and comparing the B-cell epitopes of proteins EgA31 and EgG1y62 via the software IEDB and ABCpred, we obtained the table that appeared at the same time in both web-servers, and the advantages of B-cell epitopes are shown in Table 1.

### 3.3. Construction of CVE31-162

**Figure 1:**
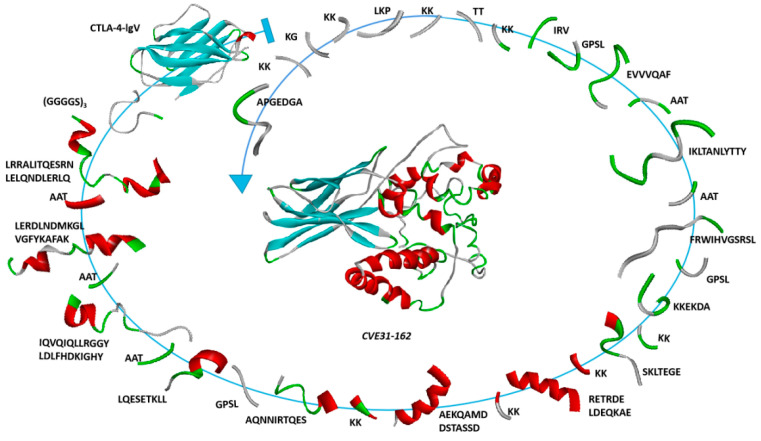
Constructive diagram of the final vaccine. The green color represents the b-turn, the red color represents the α-helix, the blue color represents the b-sheet, and the gray color represents random coil.

When building the multi-epitope vaccine *CVE31-162*, we connected the core epitopes of each antigen through linker proteins in the order of CTLA-4lgV-TEs (EgA31) -BEs (EgA31) -TEs (EgG1y62) -BEs (EgG1y62). Its connection sequence was HVAQPAVVLASSRGIASFVCEYASPGKATEVRVTVLRQADSQVTEVCAATYMMGNELTFLDDSICTGTSSGNQVNLTIQGLRAMDTGLYICKVELMYPPPYYLGIGNGTQIYVIGGGGSGGGGSGGGGSLRRALITQESRNLELQNDLERLQAATLERDLNDMKGLVGFYKAFAKAATIQVQIQLLRGGYLDLFHDKIGHYAATLQESETKLLGPSLAQNNIRTQESKKAEKQAMDDSTASSDKKRETRDELDEQKAEKKSKLTESGEKKKKEKDAGPSLFRWIHVGSRSLAATIKLTANLYTTYAATEVVVQAFGPSLIRVKKTTKKLKPKKKGKKAPGEDGA. The construction map of vaccine *CVE31-162* is shown in Figure 1.

### 3.4. The Prediction of the Physicochemical Parameters and Structure of Vaccine CVE31-162

*CVE31-162* consists of 344 amino acids, with a molecular formula of C_1624_H_2651_N_461_O_511_S_10_ and a molecular weight of 37,131.30 Da. Its theoretical pI is 9.24, and the instability index (II) is 43.53, classifying it as an unstable protein (proteins with II < 40 are stable). The grand average of hydropathicity (GRAVY) is −0.429, indicating that *CVE31-162* is hydrophilic.

Based on the antigenicity analysis of *CVE31-162* by the VaxiJen 2.0 software, the nearest protein was UniProtKB, accession number Q9UIF8. It was defined as non-allergenic. According to the results of theAllerTOP20 software ’s allergenicity analysis of vaccine *CVE31-162*, when a parasite was selected as a model, the thresh old value was 0.5 and the overall prediction for the protective antigen was 0.6930, which belonged to the probable antigen.

**Figure 2:**
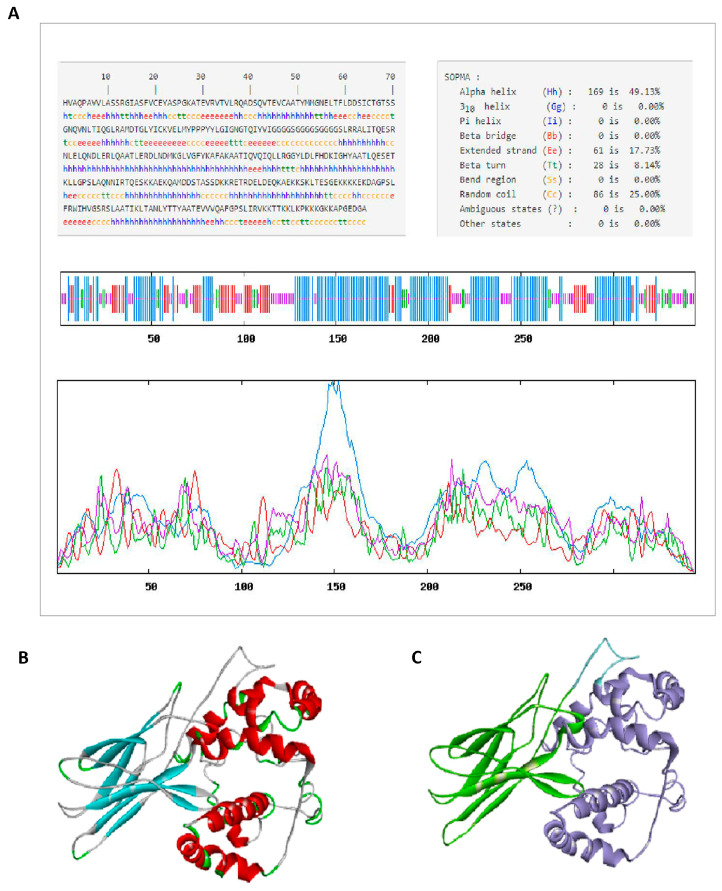
Prediction of secondary and tertiary structure: (**A**) secondary structure of *CVE31-162*; (**B**) tertiary structure for color editing based on secondary structure. (**C**) tertiary structure for color editing based on the epitope structure.

The secondary structure of *CVE31-162* was predicted by the online software SOPMA, and the results were shown in Figure 2A. They percentage composition was 49.13% alpha helix, 17.73% extended strand, 8.14% beta turn and 25.00% random coil, the red color represents the α-helix.

We used online software to predict and verify the tertiary structure of *CVE31-162.* Figure 2B is a color edit based on the secondary structure. Green represents β-turn, cyan represents β-sheet, gray represents random coil, and red represents α-helix. Figure 2C distinguishes the tertiary structure in three colors: green, blue and purple. Green represents the structure of CTLA-4lgV, purple represents the structure of the T- and B-dominant epitopes of EgA31 and EgG1Y162, and blue represents the structure of linker-(GGGGS)_3_, which connects the two. Ramachandran drawn by SWISS-MODEL was used to evaluate the three-level structural model, see Supplementary Material S1 (Figure S3).

### 3.5. Molecular Docking for CVE31-162 and B7

#### 3.5.1. Acquisition of B7-1 and B7-2 Molecules

The human B7-1/CTLA-4 and B7-2/CTLA-4 co-stimulatory complexes are available in the PDB database. Their IDs were 1I8L and 1I85, respectively, and we then edited the obtained co-stimulatory complexes with a dimer state using DS software to obtain the monomer structure models of B7-1 and B7-2.

#### 3.5.2. The Docking of CVE31-162 with B7

**Figure 3:**
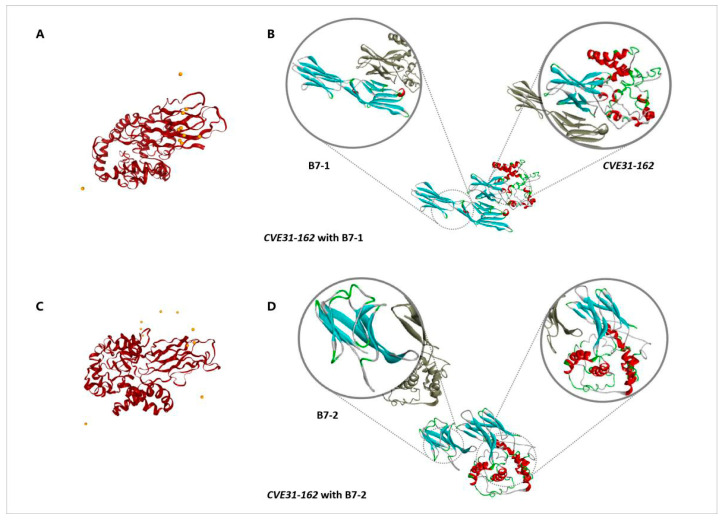
Fine docking complexes of B7-1 and B7-2 with vaccine structure. (**A**) is the top ten positions docked with B7-1. (**B**) is the top ten positions docked with B7-2. (**C**) shows the reasonable position of vaccine structure docking with B7-1. (**D**) shows the reasonable position of vaccine structure docking with B7-2.

The LZerD web server was used to simulate the molecular docking of *CVE31-162* and B7. In molecular docking, B7 was used as the receptor and *CVE31-162* as the ligand. Finally, we obtained *CVE31-162* with B7-1, and *CVE31-162* with B7-2. According to the output results of the LZerD Web Server, we chose the most reasonable docking pose from the top ten lists. The docking score and rank are displayed in Table 2. The best docking postures of *CVE31-162* and B7-1 are shown in Figure 3A,B, and the best docking postures of *CVE31-162* and B7-2 are shown in Figure 3C,D.

### 3.6. Molecular Dynamics Simulation and Principal Component Analysis

#### 3.6.1. Molecular Dynamics Simulation

**Figure 4:**
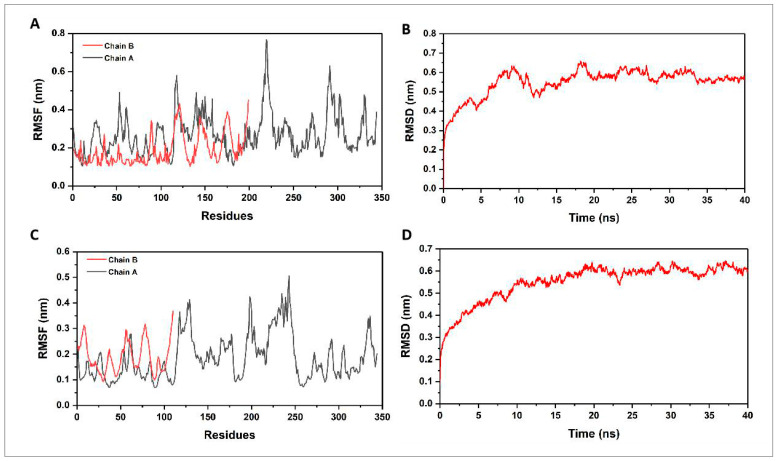
Trajectory analysis of vaccine–receptor docking complexes. (**A**,**C**) root mean square fluctuation plots, (**B**,**D**) root mean square deviation plots.

We used the gmx rmsd and gmx rmsf programs in GROMAC to obtain the RMSD (root mean square deviation) and the RMSF (root mean square fluctuation) [47,48]. The trajectory analysis of the *CVE31-162*-B7 docking complex was carried out by 40 ns. The RMSD values indicate how much the structure deviates from its initial conformation, reflecting the overall stability of the complex. For B7-1, an RMSD fluctuation between 0 and 0.65 suggests moderate stability (Figure 4B), while RMSF values between 0.1 and 0.8 indicate regions of flexibility within the protein (Figure 4A). Similarly, for B7-2, an RMSD fluctuation between 0.1 and 0.65 also suggests moderate stability (Figure 4D), with RMSF values between 0.05 and 0.5 indicating relatively lower flexibility compared to B7-1 (Figure 4C). These analyses help identify stable and flexible regions, which are crucial for understanding the dynamic behavior of the docking complex and its potential interactions.

#### 3.6.2. Principal Component Analysis

**Figure 5:**
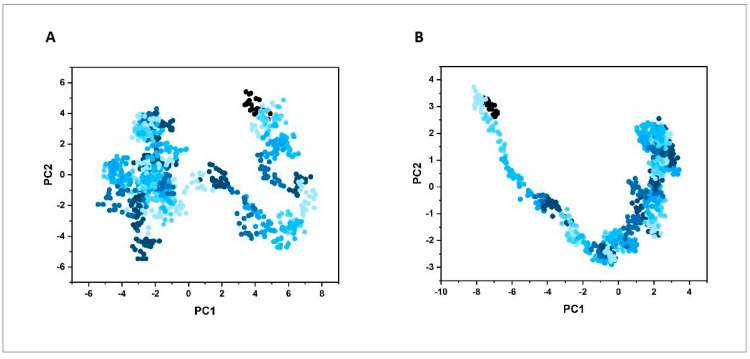
Trajectory projection of the docking complex. (**A**) B7-1-vaccine complex. (**B**) B7-2-vaccine complex. The light blue represents the time near the beginning of the simulation (such as 0 ns), and the dark blue represents the time near the end of the simulation (such as 40 ns). The overall blue gradient reflects the gradual change of protein conformation from the beginning to the end of the simulation, indicating the conformation distribution at different time points.

We presented the distribution of principal components (PC1 and PC2) for the proteins B7-1 and B7-2 in the PC phase space. The values indicate the range of conformational states these proteins can adopt during the simulation period of 0 to 40 ns. Specifically, for B7-1, the PC1 ranged from −5 to 8 nm, and the PC2 ranged from −6 to 6 nm, suggesting a wider spread and variability in the conformational landscape of B7-1 (Figure 5A). For B7-2, PC1 ranged from −9 to 4 nm, and PC2 ranged from −3 to 4 nm, indicating a more constrained conformational space compared to B7-1 (Figure 5B). These results suggest that B7-1 exhibits greater structural flexibility, potentially enhancing its ability to interact with other molecules and modulate immune responses. In contrast, B7-2 displays a more restricted range of conformations, indicating higher stability but reduced adaptability, which could differently influence its functional interactions and biological roles compared to B7-1.

### 3.7. Immune Simulation

**Figure 6:**
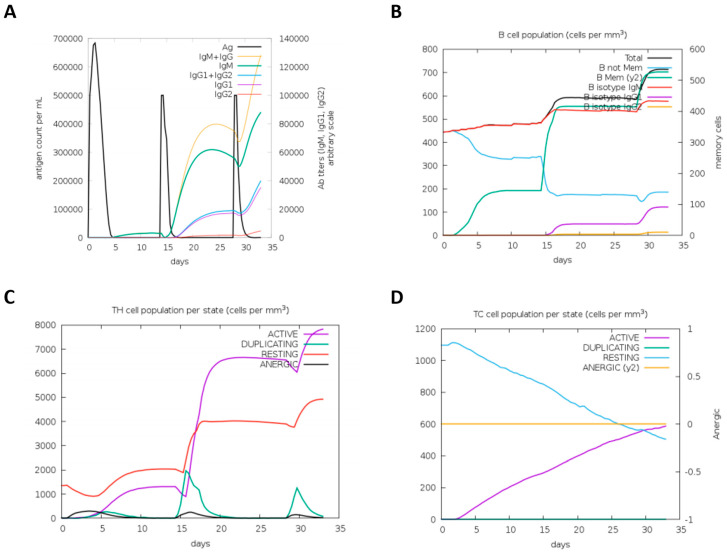
The spectrum of immune response simulation is depicted as follows: (**A**) Antibody production demonstrated a sharp increase in immune activity after three immunizations, with different immunoglobulin subtypes represented in distinct colors. (**B**) B-cell populations capable of generating various antibody subtypes following vaccination. (**C**) HTL populations at different developmental stages. (**D**) CTL populations at various stages of progression.

Immune simulation was carried out using the C-ImmSim server to simulate the immune response produced in the body when exposed to the designed vaccine. Usually, for the results of the primary immune response, the first antibody produced was mainly IgM [49]. As shown in Figure 6, after the first injection of the vaccine structure, the number of IgM increased. After the second and third injections, the levels of IgM + IgG increased significantly, and the antigen levels decreased. At the same time, the levels of IgM, IgG1 + IgG2 and IgG1 increased significantly. Two booster injections led to a rapid increase in TH cells and increased active T-cells.

### 3.8. In Vitro Experiment

#### 3.8.1. Detecting the Antigenicity of CVE31-162 by WB

**Figure 7:**
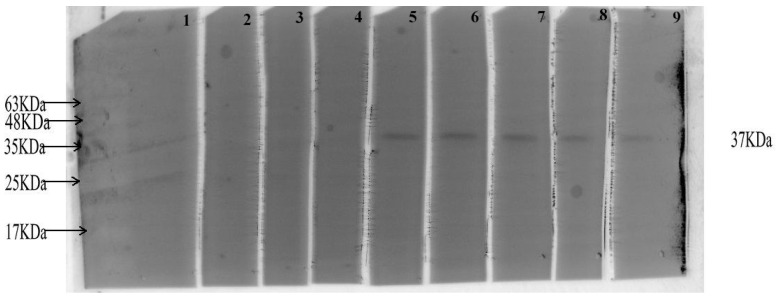
Western blotting diagram. Western blotting was used to detect the specific antibody response, with the specific antibody observed at 37 kDa. Lanes 1–4 represent the control group, while lanes 5–9 correspond to the CE group.

Western blot (WB) was used to evaluate the specific antibody against *CVE31-162* in the serum of infected patients. The results indicated that the CE group contained a specific antibody targeting the vaccine antigen, while no specific antibody was detected in the control group (Figure 7). The band images of markers and *CVE31-162* protein in Supplementary Material S1 (Figures S4 and S5).

#### 3.8.2. Assessment of T-Cell Immune Response Induced by the Vaccine

**Figure 8:**
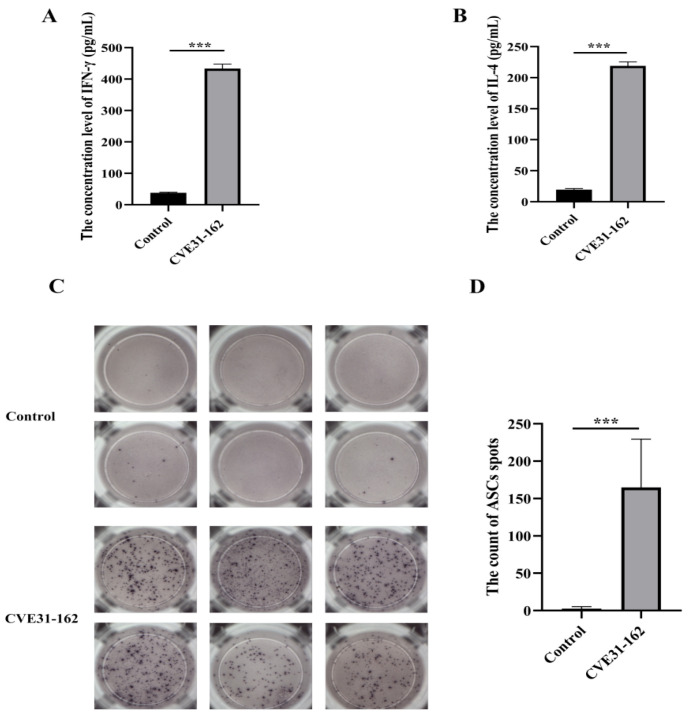
Results of the in vitro experiment: (**A**) The diagram presents a statistical comparison of IFN-γ concentration levels between the control and *CVE31-162* groups, analyzed using a two-sample *t*-test (*** *p* < 0.001). (**B**) The statistical analysis of IL-4 concentration levels between the control and *CVE31-162* groups, also conducted using a two-sample *t*-test (*** *p* < 0.001). (**C**) Representative ELISPOT spots, where blue-purple spots indicate ASCs. (**D**) Statistical analysis of ASC spot counts between the control and *CVE31-162* groups, performed using a two-sample *t*-test (*** *p* < 0.001).

Co-cultivation of PBMCs with the *CVE31-162* protein revealed a significantly higher level of IFN-γ and IL-4 in the *CVE31-162* group compared to the control group, as determined by the ELISA assay. The differences were statistically significant (*p* < 0.001), as shown in Figure 8A,B.

#### 3.8.3. Assessment of B-Cell Immune Response Induced by the Vaccine

The ELISPOT assay demonstrated that mice vaccinated with the vaccine elicited a specific B-cell response (Figure 8C). The difference in the count of specific cell spots between the *CVE31-162* group and the control group was statistically significant (*p* < 0.001, Figure 8D).

### 3.9. In Vivo Experiment

#### 3.9.1. Analysis of Specific CD4+ and CD8+ T-Cell Levels by FCM

**Figure 9:**
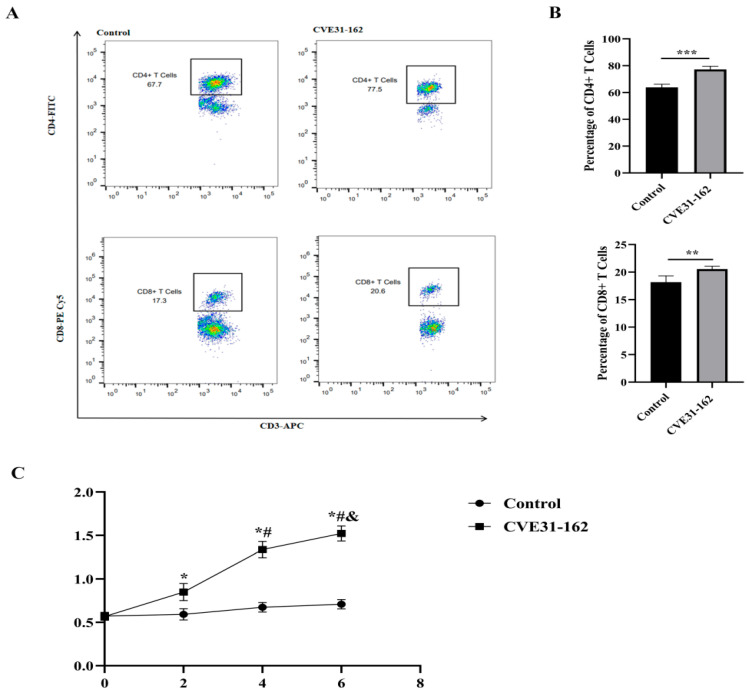
Results of the in vivo experiment. (**A**) Representative FCM strategy diagram for CD4+ and CD8+ T cells. (**B**) Statistical comparison of CD4+ and CD8+ T cell percentages between the control and *CVE31-162* groups using a two-sample *t*-test (*** *p* < 0.001, ** *p* < 0.01). (**C**) Dynamic detection of specific antibodies, where * indicates *p* < 0.01 compared to the control group, # indicates *p* < 0.01 compared to the second week within the MEV group, and & indicates *p* < 0.01 compared to the fourth week within the MEV group.

FCM was utilized to evaluate the levels of specific CD4+ and CD8+ T cells (Figure 9A). The analysis revealed that the *CVE31-162* group exhibited significantly higher levels of specific CD4+ and CD8+ T cells compared to the control group (*p* < 0.001, *p* < 0.01; Figure 9B).

#### 3.9.2. Detection of the Specific Antibody

To evaluate the antigenicity of *CVE31-162*, mice were immunized with the protein, and serum was collected to measure specific antibody levels. As shown in Figure 9C, antibody levels in the *CVE31-162* group were significantly higher than in the control group at two weeks post-first immunization (*p* < 0.01) and peaked at the sixth week after the third immunization. Additionally, serum antibody levels in the *CVE31-162* group increased significantly from baseline (0 week, *p* < 0.01) and the second week (*p* < 0.01). These findings indicate that *CVE31-162* effectively enhances antibody production and activates humoral immunity in this model.

#### 3.9.3. Detection of the Macrophages Level in Spleen and Liver with FCM

**Figure 10:**
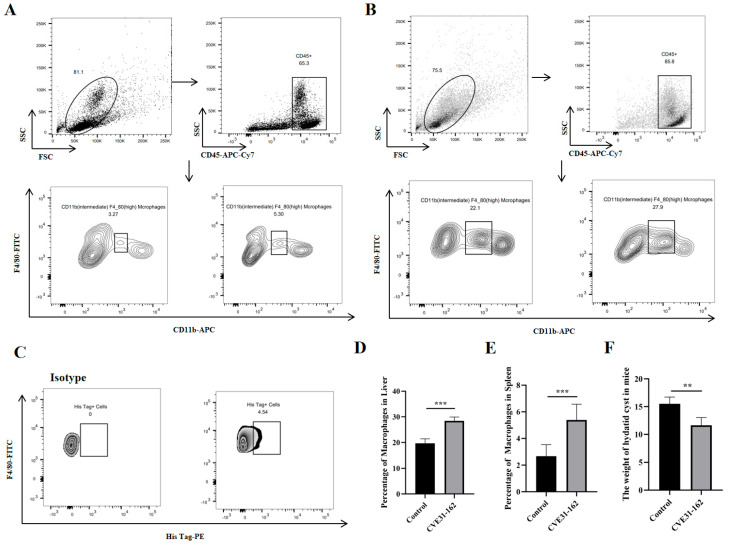
The results of the vivo experiment. (**A**) The representative FCM strategy diagram of macrophages cells in spleen. The left side represents the control group, and the right side represents the *CVE31-162* group. (**B**) This diagram is the statistical analysis of macrophages cells in the liver. The left side represents the control group, and the right side represents the *CVE31-162* group. (**C**) FCM analysis of macrophage uptake of the vaccine. (**D**) The statistical analysis of the percentage of macrophages in the liver, *** *p* < 0.001. (**E**) The statistical analysis of the percentage of macrophages in the spleen, *** *p* < 0.001. (**F**) The statistical analysis of the weight of hydatid cyst in mice, ** *p* < 0.01.

FCM was used to evaluate macrophage levels (Figure 10A,B) to assess the ability of *CVE31-162* to activate innate immunity. The results indicated significantly higher macrophage levels in the *CVE31-162* group compared to the control group (*p* < 0.001) in both the liver and the spleen (Figure 10D,E).

#### 3.9.4. Assessing the Uptake of CVE31-162 by Macrophages

In view of the strong interaction between CTLA-4 and B7 molecules, FCM was used to detect the uptake of macrophages expressing B7 molecules by vaccines. The results indicate that the proportion of His tag-positive cells is 4.54%, suggesting that the vaccine protein was successfully captured by macrophages (Figure 10C).

#### 3.9.5. Evaluation of Potential Protective Effects of CVE31-162

To evaluate the vaccine’s protective efficacy, a mouse infection model was established using *E. granulosus* protoscolices. The results showed a significant reduction in hydatid cyst weight in the immunized group compared to the control group (*p* < 0.001), as shown in Figure 10F. The immunized mice had notably lower cyst weights, indicating that the vaccine provided strong protection and effectively reduced the impact of *E. granulosus* infection.

## 4. Discussion

Vaccines represent one of the most cost-effective strategies for preventing infectious diseases [14]. The active research and development of a vaccine for echinococcosis is a crucial approach to reducing the infection rate of this disease. Multi-epitope vaccines (MEV) can stimulate a broader range of immune cells, including B-cells and various T-cell subsets, by incorporating epitopes that are specifically recognized by these cells. This results in a more potent and sustained immune response, as evidenced by the increased production of cytokines like IFN-γ and IL-4, and the generation of memory cells [50,51], as observed in our study with *CVE31-162*.

Compared with traditional vaccines, the *CVE31-162* has many another advantages. By selecting specific epitopes, MEVs can minimize the inclusion of components that might cause adverse reactions, which are sometimes associated with whole-pathogen vaccines [52]. MEVs can be tailored to include epitopes that are presented by high-frequency HLA alleles in specific populations, enhancing their efficacy in diverse demographic groups [53,54]. They consist of exogenous antigens, and their antigen epitopes must be presented to TCR by HLA-II molecules as the initial signal for T-cell activation [55]. Notably, there are regional variations in the frequency distribution of HLA alleles within the population [26]. The rural areas of Xinjiang have experienced a high incidence of echinococcosis, influenced by prevailing sanitary conditions and living habits (World Health Organization, 2015). Consequently, this study selected three high-frequency alleles—HLA-DRB1*0301, HLA-DRB1*0701, and HLA-DRB1*1501—for T-cell epitope analysis, indicating that the multi-epitope vaccine designed herein would be particularly suitable for Xinjiang, China. The design of MEV allows for the exclusion of non-essential or potentially harmful components, improving their safety profile compared to traditional vaccines [56]. The *CVE31-162* designed in this study not only contains the advantages of MEV, but also includes the molecular adjuvants CTLA-4lgV, and enhances antigen presentation by promoting the interaction between antigen-presenting cells (APCs) and T cells. This boosts the vaccine’s ability to generate a stronger and more effective immune response, particularly through the enhanced activation of T-cell immunity, which is crucial for long-term protection against *E. granulosus* infections [29,57].

In conducting this study, two software programs were utilized to screen T- and B-cell epitopes during the epitope analysis, with repeated epitopes identified as core epitopes for the construction of the multi-epitope vaccine. These effective epitopes are capable of stimulating both humoral and cellular immunity [16].We connected the selected core epitopes using linker sequences. High-quality linker sequences can ensure the complete exposure of the selected core epitopes while maintaining their antigenicity and immunogenicity [58,59]. Concurrently, the CTLA-4lgV sequence was attached to the N-terminus of the vaccine to enhance the antigen-presenting capacity of APCs [47]. The CTLA-4lgV structure exhibits a strong affinity for B7 molecules, and its fusion with antigen epitopes can significantly enhance the phagocytosis of APCs [60]. After completing the design and construction of the vaccine, we further analyzed the physical and chemical properties of the vaccine. Unstable *CVE31-162* can be rapidly degraded within antigen-presenting cells, allowing protein peptides to be swiftly presented to T cells. This process ensures a prompt response of immune cells to environmental changes. Theoretical pI and GRAVY also indicate that it was reasonable as a vaccine. According to the analysis results of antigenicity, the vaccine *CVE31-162* with a higher probability of antigens. Allergenicity analysis results showed that vaccine *CVE31-162* was a non-allergen that was safer for humans as a vaccine. The secondary structure of the vaccine protein was analyzed, and the results showed that the vaccine protein was composed of 8.14 percent beta turn and 25.00% random coil. The loose structure of beta turn and random coil can easily form epitope [39]. Therefore, the constructed vaccine had a structure that can become a good vaccine. To predict the tertiary structure of the protein, the Ramachandran results indicated that the structure was reasonable. This 3D structure would be further used in molecular docking and molecular dynamics simulation, which indicates that the two results had good reliability and validity.

The combination of antigens and CTLA-4lgV with B7 molecules can promote the presentation of APC antigens [23,61]. In order to verify the enhancement of CTLA-4lgV, we carried out molecular docking using the LZerD web server. The results showed that the simulated docking position was mainly concentrated around the CTLA-4lgV structure, which showed that the connection of the CTLA-4lgV structure can be more conducive to the presentation of APC antigens, thus improving the effectiveness of the vaccine.

According to the results of molecular dynamics simulation and PCA, the fluctuation range of the vaccine-B7 docking complex can be within a reasonable range, and smaller. The docking complex between the vaccine and the receptor has good stability [23]. It showed that the multi-epitope vaccine *CVE31-162* we built is not only a strong affinity with B7, but also a stable vaccine role, and that it was a good hydatidosis vaccine.

Immune simulation software was used to simulate cellular immunity. The injection procedure was carried out every two weeks, with a total of three injections. The immune simulation results showed that the favorable immune response lasted until the end of the immune simulation. These results showed that antibodies had good affinity with the vaccine structure, producing a certain number of memory cells, which led to an increase in the scavenging rate of antigens when exposed later. It was confirmed that the multi-epitope vaccine designed in this study had a good immune response.

This study combined in vitro and in vivo experiments to evaluate the immunogenicity and antigenicity of *CVE31-162*. Rostami et al. [62] highlighted the critical role of Th1 and Th2 immune responses in combating *E. granulosus*, noting that a shift from Th1 to Th2 reactivity may contribute to the persistence of CE. In our in vitro experiments, cellular immune function was initially assessed using ELISA.

The results indicate that the *CVE31-162* protein significantly stimulates specific Th1 and Th2 lymphocytes to produce IFN-γ and IL-4. CE is marked by the formation of a lesion microenvironment that involves the aggregation of various immune cells, particularly macrophages [63]. In the in vivo experiments, The *CVE31-162* group showed significantly higher percentages of macrophages, CD4+ T cells, and CD8+ T cells in splenocytes compared to the control group. Our results suggest that APC macrophages show good phagocytic ability towards the vaccine, which can successfully activate the innate and adaptive immunity of mice. Additionally, ELISPOT assays demonstrated that *CVE31-162* markedly enhances the production of B cells that generate specific antibodies. Mice immunized with *CVE31-162* generated specific antibodies after each injection, with levels increasing progressively with additional doses. These results indicate that *CVE31-162* effectively activates both cellular and humoral immunity in mice. Furthermore, the reduction in hydatid cyst weight suggests a protective effect against *E. granulosus* infection. In practical vaccine applications, the stability of antigens is crucial. Studies have demonstrated that antigens degrade variably across different biological environments, such as blood and lymph fluid, due to variations in protein and enzyme content and activity. This research focuses on designing a vaccine that can be efficiently presented and processed by APCs, yet it does not address the enhancement of vaccine stability. Reports indicate that the incorporation of nanoparticle delivery systems can improve vaccine stability in various biological environments, while the use of microneedle skin patches may enhance vaccine conversion efficiency [64]. In future practical applications, we will consider integrating delivery systems and engineering vaccine kinetics programming to optimize vaccine delivery efficiency and effectiveness.

## 5. Conclusions

In this study, we utilized epitope vaccines with enhanced antigenic characteristics, in conjunction with the CTLA-4lgV structure and the affinity of B7 molecules, to improve both the immunogenicity and applicability of the vaccines. The multi-epitope vaccine designed in our current research theoretically demonstrates a greater efficacy against echinococcosis. Both in vivo and in vitro experiments confirmed that the vaccine developed in this study exhibited strong antigenicity and immunogenicity.

In summary, the design of a multi-epitope vaccine *CVE31-162* targeting *E*. *granulosus*, based on the interaction of CTLA-4 and B7, is efficient and feasible.

## Figures and Tables

**Table 1:** The predictions of T- and B-cell epitopes of proteins.

Category	Software	Protein EgA31		Protein EgG1Y162	
		Position	Amino Acid Core Sequence	Position	Amino Acid Core Sequence
T-cell	IEDB	200–222	LRRALITQESRNLELQNDLERLQ	25–35	FRWIHVGSRSL
	NetMHCIIpan	580–599	LERDLNDMKGLVGFYKAFAK	52–56	IKLTANLYTTY
		263–285	IQVQIQLLRGGYLDLFHDKIGHY	89–95	EVVVQAF
		289–297	LQESETKLL		
B-cell	IEDB	20–29	AQNNIRTQES	3–5	IRV
	ABCpred	171–184	AEKQAMDDSTASSD	19–20	TT
		233–235	RETRDELDEQKAE	82–84	LKP
		399–406	SKLTESGE	96–97	KG
		492–497	KKEKDA	112–118	APGEDGA

**Table 2:** Final model score.

	GOAP Score	GOAP Rank	DFIRE Score	DFIRE Rank	ITScore Score	ITScore Rank	Ranksum Score
B7-1	−45,146.97	64	−40,241.33	73	−21,234.99	134	271
B7-2	−29,058.07	76	−33,083.35	712	−17,371.30	154	942

## Data Availability

The original contributions presented in this study are included in the article/Supplementary Material. Further inquiries can be directed to the corresponding author(s).

## Supplementary Materials

The following supporting information can be downloaded at: https://www.mdpi.com/article/10.3390/vaccines12121440/s1, Figure S1. Transmembrane Domains of Proteins EgA31 and EgG1y62. (A) Prediction of EgA31 transmembrane domains of *Echinococcosis*. (B) Prediction of EgG1y62 of Echinococcosis. Figure S2. The Signal peptide of Proteins EgA31 and EgG1y62. (A) Prediction of EgA31 signal peptide of *E. granulosus*. (B) Prediction of EgG1y62 signal peptide of *E. granulosus*. Figure S3. The bands figure with marker. Figure S4. The bands figure with CVE31-162 protein.
