# Supplementary material for: Enhanced Immune Response Against Echinococcus Granulosus Through a CTLA-4/B7 Affinity-Based Vaccine
Source: Vaccines (Basel). 2024 Dec 20;12(12):1440. doi: 10.3390/vaccines12121440 (PMC11680267; doi:10.3390/vaccines12121440)
Supplement: Supplementary file 1 [file vaccines-12-01440-s001.zip › vaccines-3319824-supplementary.pdf]

The Amino Acid Sequences of Proteins

The accession of Protein EgA31 was AAC21558.1. The amino acid sequence was TRPQRKKNEYEDLELQLENAQNNIRTQESNCRRLSLEHMKALEEIKMKQITM EGLETKITELIQRNEDLTKEALNTKNVESSNREEVLLSKIKSLEKTAKHLHIVL KEEKHYNNQLKEEIDEIRKENLATLQTRLNEIFQKESEMNSERKALNMRIMAL EAENERLRISAAEKQAMDDSTASSDGIYFAIEEERKKSAELRRALITQESRNLE LQNDLERLQRETRDELDEQKAEIEKLHKELVGADNSKTTVQSIRNEMRGIQV QIQLLRGGYLDLFHDKIGHYKEKLQESETKLLELQGTHDQTKRIHDVEKDKL AQELKYMEKQINLCSNENNKLKDALASMENQITGLLNGNELLKKKIEMLETR AETKEINTKLENLLKTETQELLTTIKDLDSKLTESGEEVKELKKQLEKAEKQIR DAEVLTDEKNKIIEEMKKTINKLSETKKEVEDKNSELRKASIQKQSLIEEKEIL GRQLELKDTIISAMKKEKDALRHELHEMRDTINTLTEKIATIKIPEPLPMKPIEIP KPLQKEGVNKAMENEIQTYKDTIKTLKDEIFDKSRVINENQVIIKQLERDLND MKGLVGFYKAFAKKK.

The accession of Protein EgG1Y162 was BAH28839.1. The amino acid sequence was MVLRFCLILLATSVIAEEIRVDPELMAKLTKELKTTLPEHFRWIHVGSRSLELG WNATGLANLHADHIKLTANLYTTYVTFKYRNVPIERQKLTLEGLKPSTFYEVV VQAFKGGSQVFKYTGFIRTLAPGEDGADRASGFALIFAMAGLLLLT.

The accession of Protein CTLA-4 was AAD00698.1, where amino acids 2 through 115 is CTLA-4lgV. The amino acid sequence of CTLA-4lgV was HVAQPAVVLASSRGIASFVCEYASPGKATEVRVTVLRQADSQVTEVCAATYM MGNELTFLDDSICTGTSSGNQVNLTIQGLRAMDTGLYICKVELMYPPPYYLGI GNGTQIYVI.

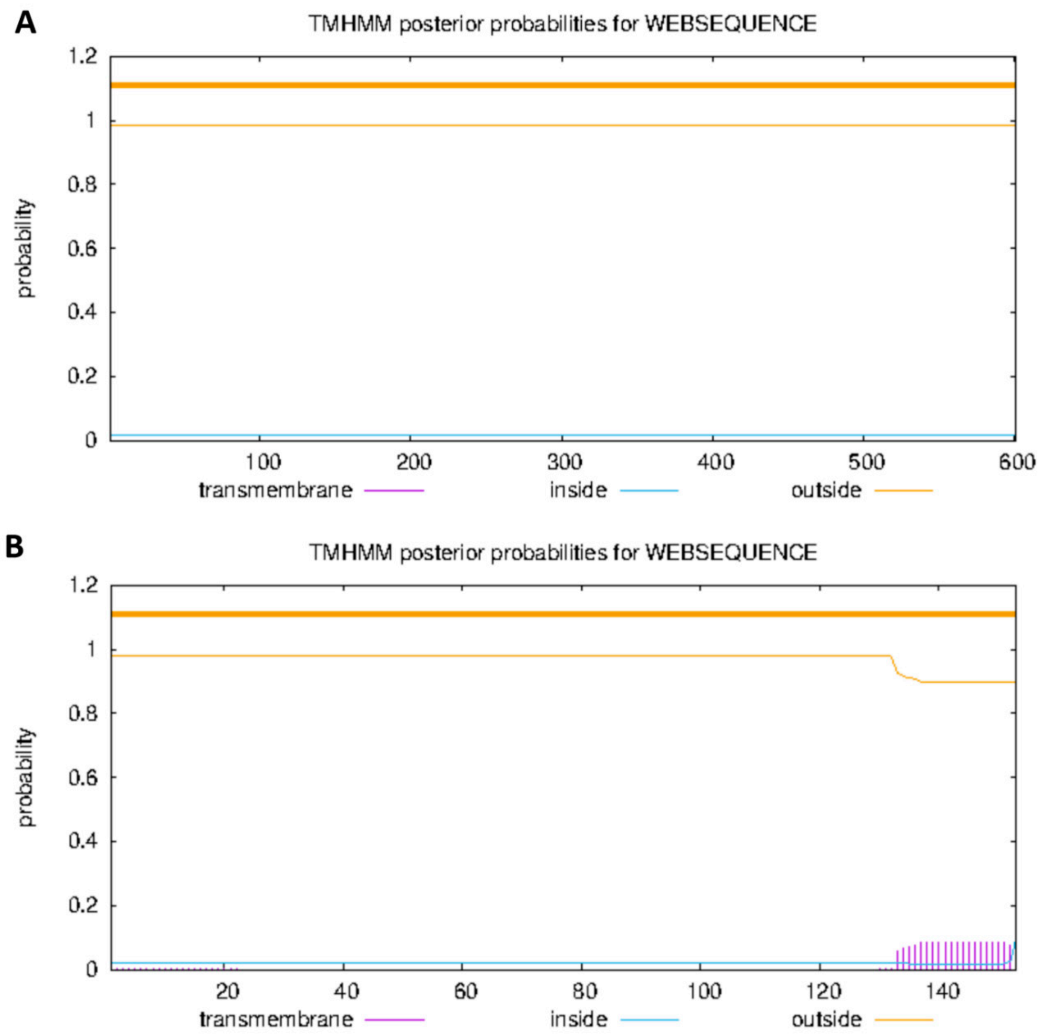

Figure S1. Transmembrane Domains of Proteins EgA31 and EgG1y62. (A) Prediction of EgA31 transmembrane domains of *Echinococcosis*. (B) Prediction of EgG1y62 of *Echinococcosis*.

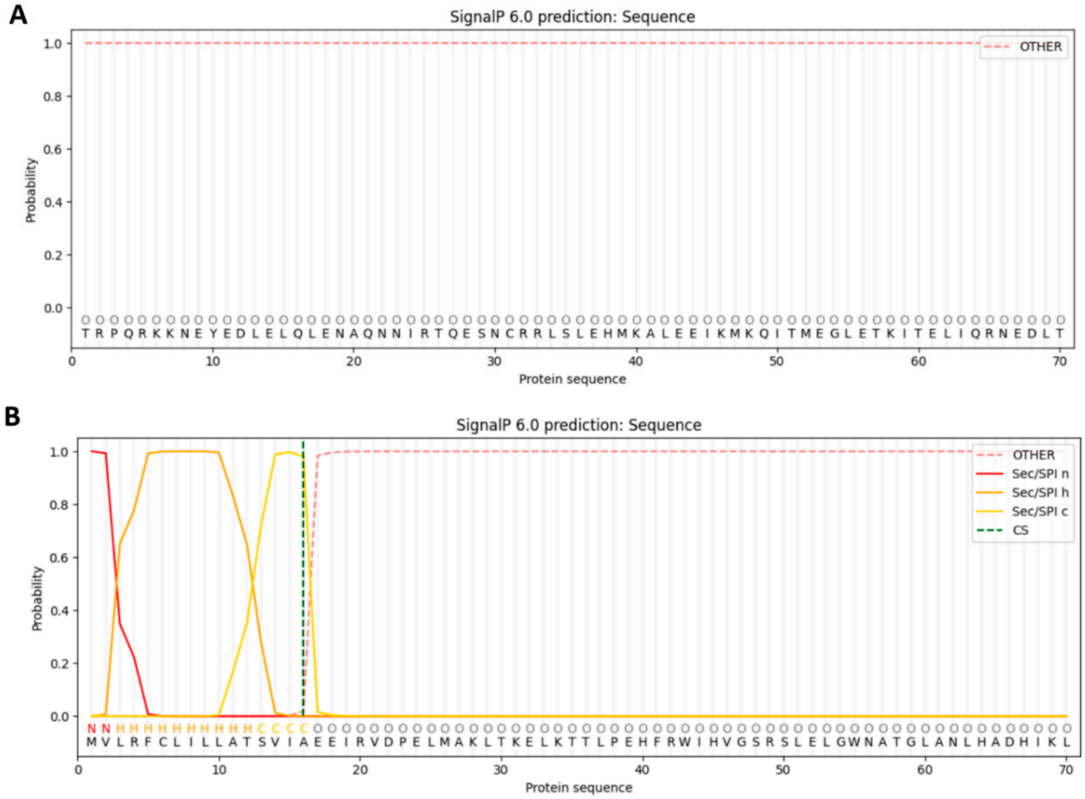

Figure S2. The Signal peptide of Proteins EgA31 and EgG1y62. (A) Prediction of EgA31 signal peptide of *E. granulosus*. (B) Prediction of EgG1y62 signal peptide of *E. granulosus*.

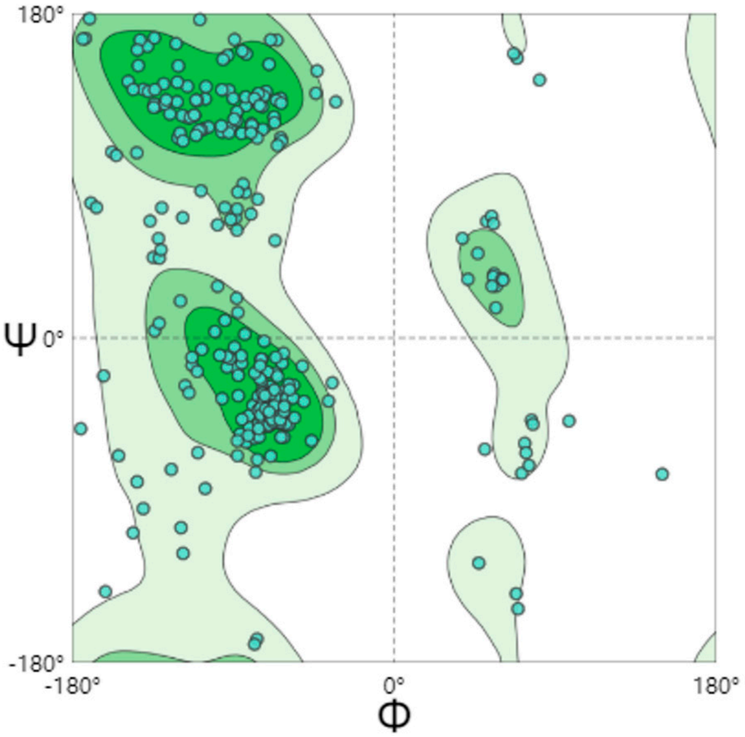

Figure S3. The Ramachandran figure was drawn by SWISS-MODEL. Most of the structures were in Ramachandran favored region, which means that the tertiary structure of the vaccine is reasonable.

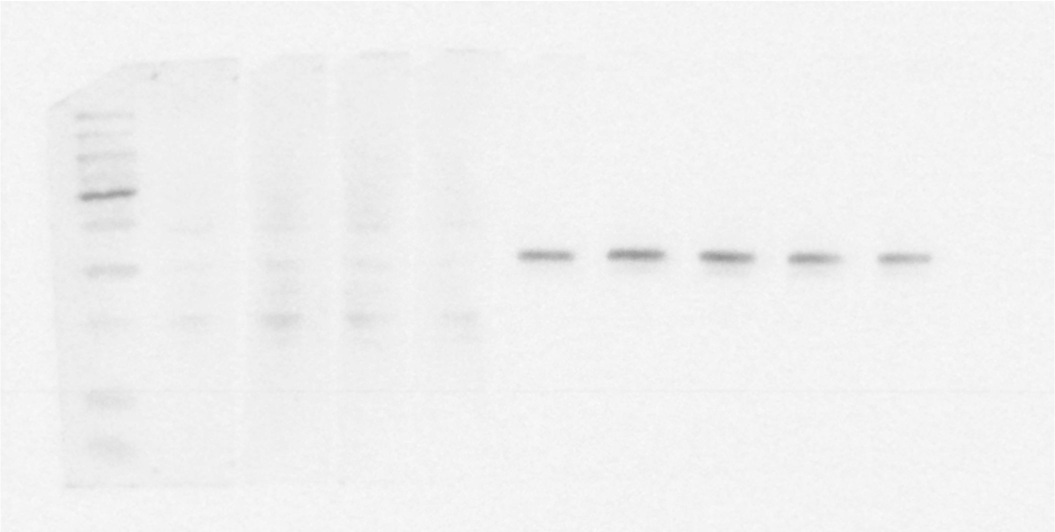

Figure S4. The bands figure with markers.

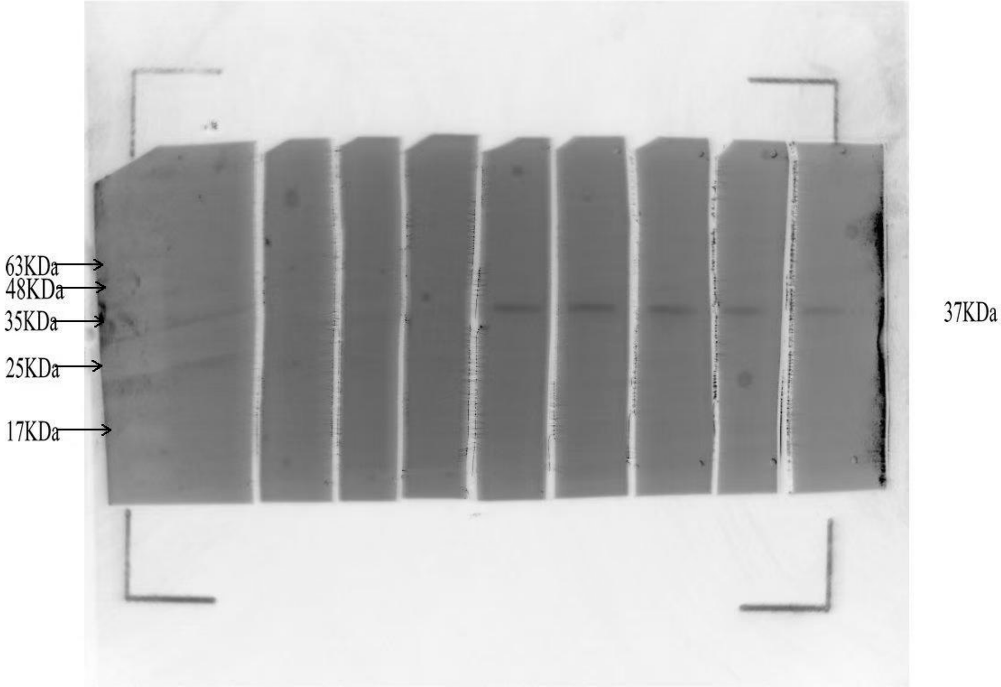

Figure S5. The bands figure with CVE31-162 protein.
